# Vertical Distribution Mapping for Methane Fugitive Emissions Using Laser Path-Integral Sensing in Non-Cooperative Open Paths

**DOI:** 10.3390/s24041307

**Published:** 2024-02-18

**Authors:** Di Wang, Yushuang Li, Yu Pu, Yan Lv, Mingji Wang, Hui Yang, Xuefeng Zhao, Dong Li

**Affiliations:** 1 School of Physics and Electronic Engineering, Northeast Petroleum University, Daqing 163318, China; wangdinepu@stu.nepu.edu.cn (D.W.);; 2 Heilongjiang Provincial Key Laboratory of Thermal Utilization and Disaster Reduction of New Energy in Cold Regions, Northeast Petroleum University, Daqing 163318, China; 3 Office of Science, Quanzhou University of Information Engineering, Quanzhou 362008, China; 4 Heilongjiang Institute of Metrological, Verification, and Testing, Harbin 150028, China; 5 Daqing Oilfield Co., Ltd., Daqing 163453, China

**Keywords:** methane fugitive emissions, laser absorption spectroscopy, optical remote sensing, tomographic algorithm, diffusion distribution

## Abstract

Observing the vertical diffusion distribution of methane fugitive emissions from oil/gas facilities is significant for predicting the pollutant’s spatiotemporal transport and quantifying the random emission sources. A method is proposed for methane’s vertical distribution mapping by combining the laser path-integral sensing in non-non-cooperative open paths and the computer-assisted tomography (CAT) techniques. It uses a vertical-plume-mapping optical path configuration and adapts the developed dynamic relaxation and simultaneous algebraic reconstruction technique (DR-SART) into methane-emission-distribution reconstruction. A self-made miniaturized TDLAS telemetry sensor provides a reliable path to integral concentration information in non-non-cooperative open paths, with Allan variance analysis yielding a 3.59 ppm·m sensitivity. We employed a six-indexes system for the reconstruction performance analysis of four potential optical path-projection configurations and conducted the corresponding validation experiment. The results have shown that that of multiple fan-beams combined with parallel-beam modes (MFPM) is better than the other optical path-projection configurations, and its reconstruction similarity coefficient (ε) is at least 22.4% higher. For the different methane gas bag-layout schemes, the reconstruction errors of maximum concentration (*γ_m_*) are consistently around 0.05, with the positional errors of maximum concentration (*δ*) falling within the range of 0.01 to 0.025. Moreover, considering the trade-off between scanning duration and reconstruction accuracy, it is recommended to appropriately extend the sensor measurement time on a single optical path to mitigate the impact of mechanical vibrations induced by scanning motion.

## 1. Introduction

Methane is a typical identification constituent of hazardous substances from various oil and natural gas systems. Methane emissions from energy activities contribute to roughly 20% of anthropogenic release, with the oil and gas sector being the primary contributor [1,2]. The methane emission sources include, but are not limited to, equipment and pipeline leaks, evaporation and flashing losses, venting, flaring, incineration, and accidental releases. The Environmental Defense Fund (EDF) reported that the actual methane emissions volume of oil and gas supply chain is approximately 60% higher than estimated by the U.S. Environmental Protection Agency (EPA) [3]. More recently, an investigation by Brouwer’s team has shown that fugitive emissions from field production account for 29.64% of the total natural gas system, with continuous micro-leakage from gathering and boosting facilities being the main contributors [4]. Typically, fugitive emissions tend to suffer from minuteness in flow rate and concentration, and are more difficult to prevent. Methane fugitive emissions cast doubt on the climate benefits attributed to natural gas when compared to oil and coal, primarily due to methane’s global warming potential (GWP), which is 25 to 34 times greater than that of carbon dioxide [5,6,7,8]. Additionally, from an energy safety standpoint, it is important to consider factors such as energy wastage, as well as the risk of fire and explosions resulting from methane accumulation [9,10].

Direct measurements of methane fugitive emissions from oil and gas facilities play a crucial role in improving existing emission models, formulating accurate emission inventory, and evaluating the feasibility and suitability of methane mitigation initiatives. However, precisely characterizing methane fugitive emissions in oil/gas facilities is a demanding measurement technique within conventional pollution monitoring and control strategies [11]. Laser spectroscopy techniques, such as tunable diode laser absorption spectroscopy (TDLAS) [12,13,14], quartz-enhanced photoacoustic spectroscopy (QEPAS) [15,16,17], light-induced thermoelastic spectroscopy (LITES) [18,19,20], etc., have garnered significant development in the field of trace methane detection in recent years due to their advantages of high sensitivity, precision, and non-contact measurement capabilities. Ye et al. presented a highly sensitive methane-detection system based on beat frequency QEPAS technology, which achieves a minimum detectable limit of 28.35 ppm for methane with an integration time of 114 s [15]. Yufei Ma’s team has successively utilized QEPAS and LITES to achieve ultra-sensitive detection of methane at ppb level concentration [17,18]. Although QEPAS and LITES can achieve high sensitivity-detection performance at low costs, they face challenges when utilized for open path telemetry. As the distance between the QTF and retro-reflection objects increase, both the electromagnetic radiation pressure and resonance amplitude experience an exponential decrease. This limitation hinders its extension to field applications involving non-cooperative targets.

Open path tunable diode laser absorption spectroscopy (OP-TDLAS) is a predominant non-contact method for detecting methane leaks [21,22,23,24]. Frish et al. devised a portable OP-TDLAS sensor capable of discerning methane concentrations within the ppm·m range at a 30 m detection distance [25]. Employing portable laser methane detectors allows for the flexible identification of pronounced and sustained leaks at anticipated locations. However, intermittent fugitive emissions may be present during leak-detection plans that may be omitted from the investigative area. Zhang et al. reported an OP-TDLAS field-deployable method for in situ methane monitoring on oil and gas well pads, and demonstrated the efficacy of the single-sensor determination of emission sources by combining the simultaneous wind velocity measurements and the angle-of-arrival algorithm [26]. However, the conventional OP-TDLAS technique can only retrieve the path-integral concentration along a single laser beam within the emission area, without monitoring the gas diffusion distribution from fugitive emissions. The monitoring of methane-emission-concentration distribution for oil/gas facilities can assist regulators in their real-time understanding of the temporal variability of emission sources and the spatial transport regularity of pollutant diffusion. This information provides a basis for formulating appropriate methane-emission-control measures.

Range-resolved differential absorption Lidar (DIAL) measurements offer a method to map and quantify the plume distribution of methane fugitive emissions. Innocenti et al. utilized the DIAL technique and a vertical scanning strategy to measure the spatial distribution of methane plumes, enabling the spatially separate and independent quantification of emissions from different sources [27]. Robinson et al. employed the IR-DIAL system to quantify the emissions from different areas of the landfill and identify and map the localized emission sources around the site [28]. Range-resolved DIAL achieves spatial resolution by capturing atmospheric light scattering at varying distances. However, the exceptionally faint echoes pose challenges, necessitating increased laser power, larger optical components, and specialized detectors, thus increasing system complexity and cost. The integration of the OP-TDLAS path-integral sensing conjunction with computer-assisted tomography (CAT) techniques has emerged as a promising method for the gas-distribution mapping of methane emissions and diffusions. Du et al. proposed a combined method using multi-path OP-TDLAS and CAT technologies to derive the methane-concentration distribution for the large-scale area source emissions [29]. Thoma et al. described the utilization of multi-path OP-TDLAS for acquiring concentration-distribution data for methane fugitive emissions from a bioreactor side hill [30]. While numerous studies have explored the application of TDLAS-CAT technology in reconstructing the concentration distribution of methane emissions, these reports have commonly employed retro-reflectors associated with laser emission. Although OP-TDLAS systems employing optical reflectors can achieve emission monitoring over a large-scale area, they may not be suitable for facility- or site-scale scenarios. This is because the laser beam propagation is defined by the on-site layout [31]. The installation of reflectors necessitates the consideration of obstacle positions, thereby limiting the optimal strategy of scanning schemes and optical path density. Li et al. employed computational fluid dynamics methods to conduct research on tomographic reconstruction using TDLAS technology for indoor pollutant distribution under non-cooperative target conditions. They quantitatively analyzed the impact of the laser-emitter placements and optical path densities on the concentration field reconstruction quantitatively [32]. However, the study did not implement specific experimental measures to verify the effectiveness of the gas-distribution mapping using the TDLAS senor under non-cooperative targets.

In this work, we propose a method for vertical gas-distribution mapping using laser path-integral sensing in non-cooperative open paths, specifically focusing on methane fugitive emissions from oil/gas-facility scenarios. A miniaturized near-IR TDLAS telemetry sensor is developed to achieve ppm sensitivity and provides path-integral concentration information for the tomographic reconstruction of methane-emission distribution under non-cooperative open path conditions. An improved tomographic algorithm, namely the dynamic relaxation and simultaneous algebraic reconstruction technique (DR-SART), is presented. The reconstruction effects of methane-diffusion distribution using four optical path-projection configurations are detailed and investigated based on the six evaluation indexes. Experimental validation is conducted for the optimal optical path configuration, and we quantitatively analyze the reconstruction quality under different methane-concentration distributions. Furthermore, an in-depth investigation is carried out on the impact of the scanning duration of optical path configuration on the reconstruction error of concentration distribution.

## 2. Methodology

### 2.1. Key Approach of TDLAS Methane Laser Telemetry Sensing

#### 2.1.1. Methane Concentration Inversion Based on Harmonic Conjoint Analysis

The core mechanism of TDLAS methane laser telemetry is the Beer-Lambert Law [33] and the transmitted laser intensity, while the laser beam passing through a methane cloud can be expressed as follows:

(1) $$I_{t}\left(v\right)=I_{0}\left(v\right)\exp\left[-kL\right]=I_{0}\left(v\right)\exp\left[-PSX\varphi L\right]$$

where *I*_t_ and *I*_0_ are the transmitted and incident laser intensities, respectively, and *v* is laser frequency; *k* is methane spectral absorption coefficient; *L* is optical path length; *P* is methane gas pressure; *X* is methane gas concentration; *S* is transition line-strength of methane molecule; *φ* is line-shape function.

Direct absorption spectroscopy (DAS) technology is the most straightforward method to derive concentration information from absorbance measurements. However, in the scenario of remote detection with non-cooperative targets, the accuracy and sensitivity of DAS sensors will be drastically deteriorated. The Wavelength Modulation Spectroscopy (WMS) technique, employing harmonic detection, enables absorbance measurements within the range of 10^−5^ to 10^−6^ [34]. This represents an enhancement of 2 to 3 orders of magnitude compared to DAS. An important advantage of WMS is to shift the detection to higher frequencies, at which the laser excess noise (1/f noise) is reduced [35]. Therefore, WMS technique is employed to obtain the remote-sensing information of methane concentration in non-cooperative target conditions. The basic work of the WMS technique is to modulate the laser injection current with a high frequency sinusoidal signal and demodulate the harmonic signals [36]. In the process of methane remote detection by using TDLAS technique, the non-cooperative targets are usually natural object surfaces such as floors, walls, and pipe walls, etc. The reflective properties of backscattered surface and nonlinear fluctuations of laser intensity will affect the measurement results. Therefore, the concentration inversion method with the first harmonic (1*f*)-normalized second harmonic (2*f*) signal is commonly used in the WMS technique to eliminate these adverse interferences. The extraction of the harmonic signals from the photodetector is realized by using the fast Fourier transform method in this work. The resulting equations for the X and Y components of the 1*f* and 2*f* signals are given by:

(2) $$\begin{array}{l} R_{1f}={\left(X_{1f}^{2}+Y_{1f}^{2}\right)}^{1/2}=\\ \frac{\eta G{\bar{I}}_{0}}{2}{\left\{{\left[H_{1}+i_{1}(1+H_{0}+\frac{H_{2}}{2})\cos\psi_{1}\right]}^{2}+{\left[i_{1}(1+H_{0}-\frac{H_{2}}{2})\sin\psi_{1}\right]}^{2}\right\}}^{1/2} \end{array}$$

(3) $$\begin{array}{l} R_{2f}={\left(X_{2f}^{2}+Y_{2f}^{2}\right)}^{1/2}=\\ \frac{\eta G{\bar{I}}_{0}}{2}{\left\{{\left[H_{2}+\frac{i_{1}}{2}(H_{1}+H_{3})\cos\psi_{1}\right]}^{2}+{\left[\frac{i_{1}}{2}(H_{1}-H_{3})\sin\psi_{1}\right]}^{2}\right\}}^{1/2} \end{array}$$

where *η* is reflectivity of non-cooperative targets; *G* is optical-electrical gain of the detection system; *I*_0_ is the average laser intensity at the center frequency; *i*_1_ is linear intensity modulation amplitude; *ψ*_1_ is linear intensity modulation phase shift; *H*_n_ is the n-th-order Fourier series expansion coefficient of spectral absorption coefficient, *H*_1_ = 0, *H*_0_, *H*_2_ << 1, thus:

(4) $$R_{1f}=\frac{\eta G{\bar{I}}_{0}}{2}i_{1}$$

(5) $$R_{2f}=\frac{\eta G{\bar{I}}_{0}}{2}H_{2}$$

The 1*f*-normalized 2*f* signal is now described by:

(6) $$\begin{array}{l} S_{2f/1f}=\frac{R_{2f}}{R_{1f}}=\frac{H_{2}}{i_{1}}\\ =\frac{PXLS}{\pi i_{1}}\int_{-\pi }^{\pi }\varphi (\bar{v}+a\cos(\omega t))\cos(2\omega t)d\omega t \end{array}$$

where *v* is the center frequency of the laser wavelength and *a* is the modulation depth.

It can be seen from Equation (6) that the WMS-2*f*/1*f* method effectively eliminates the influence of the light-intensity fluctuation on the methane remote-sensing results, which greatly improves the reliability performance of the methane laser sensor in the open-path environment. While using WMS-2*f*/1*f* in long-term measurement processes, the operation-temperature fluctuations and other random interferences will cause waveform distortion in harmonics signals. A distorted harmonic template-matching (DHTM) algorithm has been proposed to ensure the stability of indicating values during long-term monitoring processes in our previous work [37].

#### 2.1.2. TDLAS Telemetry Sensor Configuration

**Figure 1:**
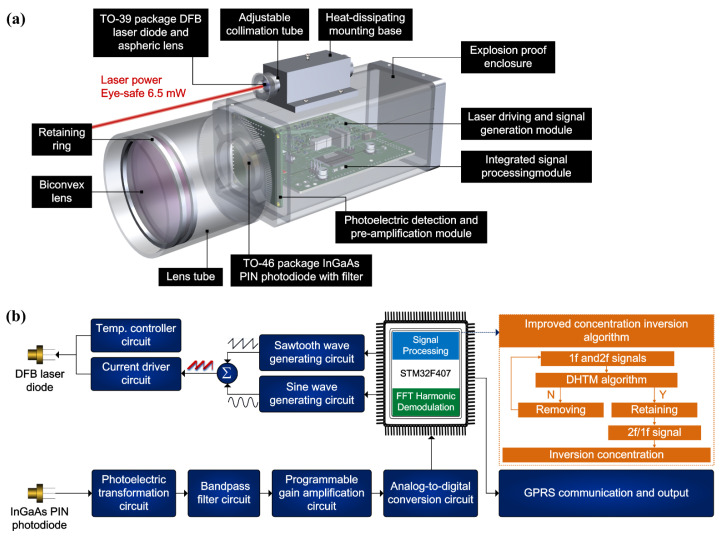
(**a**) The panorama of the self-developed portable and miniaturized TDLAS telemetry sensor (length 165.8 mm, width 56 mm, and height 68.3 mm). (**b**) The circuit and algorithm procedure of laser telemetry sensor.

The self-developed portable and miniaturized TDLAS telemetry sensor is shown in Figure 1a, which consists of a sophisticated combination of optical, mechanical, and electrical components.

The light source utilized in this telemetry sensor is a distributed feedback (DFB) laser diode (EP1654-DM-TP39, Eblana Photonics Ltd., Dublin, Ireland) enclosed in a TO-39 package. The DFB laser diode chosen for this application exhibits a central output wavelength of 1653.7 nm and a standard power output of 6.5 mW. The divergent optical radiation emanating from laser diode is collimated into a parallel beam via an aspheric lens. An N-BK7 biconvex lens, characterized by a diameter and focal length both equal to 50 mm, is positioned immediately beneath the adjustable collimation tube. Its primary function is to capture the backscattered light from non-cooperative targets. The faint laser backscattered light, carrying information pertaining to methane concentration, is focused into the TO-49 -packaged InGaAs PIN photodiode (LSIPD-L2, Beijing Lightsensing Technologies Ltd., Beijing, China). An IR bandpass filter (FB1750-500, Thorlabs Inc., Newton, NJ, USA) is positioned at the forefront of the photodiode to selectively exclude undesired ambient stray radiation. Subsequently, the optical signal is transferred into an electrical signal, intended for use within the subsequent circuit processing system. The circuit and algorithm procedures are shown in Figure 1b. The electrical unit comprises three distinct circuit modules: a module responsible for laser driving and signal generation, a photoelectric detection and pre-amplification module, and an integrated signal-processing module facilitating harmonic demodulation, concentration inversion, GPRS communication, and power supply functions. The precision of TDLAS is notably influenced by the harmonic signal waveform. Consequently, the DHTM algorithm is employed to identify and extract the undistorted harmonic signals for the concentration-inversion process.

### 2.2. Gas-Distribution Mapping Using Laser Path-Integral Concentration

#### 2.2.1. Dynamic Relaxation and Simultaneous Algebraic Reconstruction Algorithm

**Figure 2:**
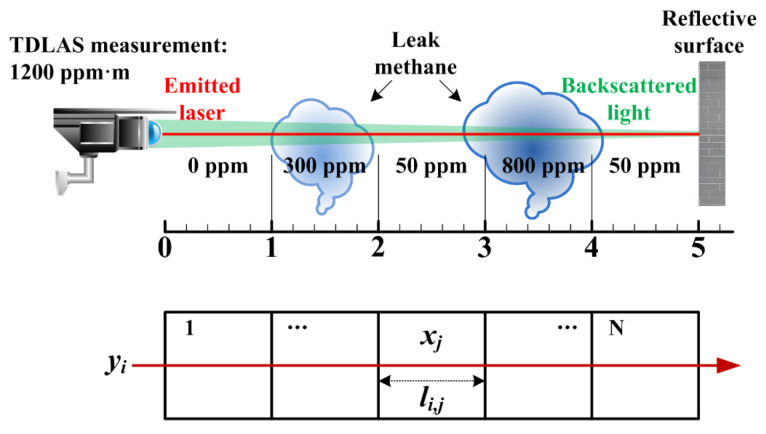
Detection mode of single laser beam is adopted to measure the non-uniform methane-concentration field. Illustrating with an example, the laser beam travels for 5 m and the methane concentration along the beam path is incongruent. The indicating value of TDLAS measurement in this case is 1200 ppm·m = (0 ppm × 1 m + 300 ppm × 1 m + 50 ppm × 1 m + 800 ppm × 1 m + 50 ppm × 1 m).

The laser telemetry sensing based on TDLAS technology essentially belongs to one-dimensional line-of-sight (LOS) measurements along the direction of laser beam transmission, and the measurement result obtained is the path-integral concentration (ppm·m). As shown in Figure 2, Trincavelli et al. [38] elucidated the classical measurement principle of path-integral concentration for TDLAS telemetry sensors. It is worth noting that gas molecules will doubly absorb the photons of the emitted laser and backscattered light. This implies that the effective absorption path length will be associated with gas absorbance including the path length of scattered light. However, the path length of scattered light is unknown in practical measurements. Although the characterization of path-integral concentration relies solely on the distance along the emitted laser path (i.e., detection distance), the dual absorption has already been considered in the calibration process of system measurement. Moreover, the gas-diffusion region can be discretized into a uniform grid of square cells. It is assumed that the methane concentration in each cell is constant [39], so the TDLAS measurement alone for the single laser beam path is expressed as follows:

(7) $$y_{i}=\sum_{j=1}^{N}l_{i,j}\cdot x_{j}$$

where *y_i_* is the path-integral concentration measured by the laser beam *i*, *N* is the number of square cells of the uniform grid, *l_i_*_,*j*_ is the absorption path length of the laser beam *i* in the *j*-th cell and *x_j_* is the averaged methane concentration in cell *j*.

**Figure 3:**
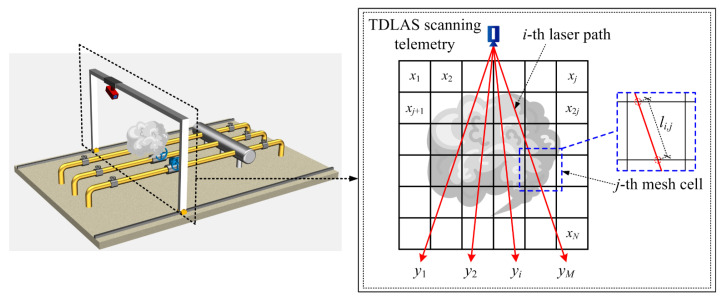
Schematic of scanning laser path-integral concentration and the discretization configuration for methane-diffusion area.

Figure 3 demonstrates a square methane-diffusion area of interest that is hypothetically discretized into *N* identical cells. Scanning the area to be measured using a TDLAS telemetry sensor, a set of path-integral concentrations *y_i_* from *M* laser beam paths in multiple angles or intervals are acquired. Therefore, the whole region can be described by the vector ***Y*** (***Y*** ∈ **R**^*M* × 1^), and the mean methane concentrations in each cell can form a column vector ***X*** (***X*** ∈ **R**^*N* × 1^). Their relationship can be written as

(8) Y=L X

where the ***L*** (***L*** ∈ **R**^*M* × *N*^), called the weight coefficients matrix, contains the geometrical path length of each laser beam passing through *M* cells.

Generally, Equation (8) can be compactly rewritten in the following matrix expression:

(9) $$\left[\begin{array}{c} y_{1}\\ y_{2}\\ \vdots \\ y_{M} \end{array}\right]=\left[\begin{array}{cccc} l_{1,1} & l_{1,2} & \cdots & l_{1,N}\\ l_{2,1} & l_{2,2} & \cdots & l_{2,N}\\ \vdots & \vdots & & \vdots \\ l_{M,1} & l_{M,2} & \cdots & l_{M,N} \end{array}\right]\left[\begin{array}{c} x_{1}\\ x_{2}\\ \vdots \\ x_{N} \end{array}\right]$$

The matrix ***L*** can be ascertained in advance from the geometric distribution of the optical path projection. The path-integral concentration *y_M_* is obtained with TDLAS telemetry sensor measurements. Here, the local averaged methane concentrations *x_j_* at each grid can be calculated by solving the linear equations based on tomographic algorithms. However, ill-conditioned equation problems will be introduced in the process of tomographic reconstruction [40]. The iterative algorithm is a serviceable means to tackle the ill-conditioned inversion problem. The simultaneous algebraic reconstruction technique (SART) is derived from the algebraic reconstruction technique (ART) tomographic algorithm. In contrast to the ART algorithm, the SART algorithm simultaneously updates the corrections of all laser paths passing through a certain grid during the iterative reconstruction calculation [41]. This improvement can reduce the influence of severe artifacts and random noise, while increasing the quality of reconstruction results. Therefore, in this work, the linear equations are solved iteratively by operating SART tomographic algorithms as follows:

(10) $$x_{j}^{\left(k+1\right)}=x_{j}^{\left(k\right)}+\frac{\lambda }{\sum_{i=1}^{M}l_{i,j}}\sum_{i=1}^{M}\left(\frac{y_{i}-\sum_{j=1}^{N}l_{i,j}x_{j}^{\left(k\right)}}{\sum_{j=1}^{N}l_{i,j}}l_{i,j}\right)(i=1,2\ldots ,M;\;j=1,2\ldots ,N)$$

In Equation (10), *k* is the iteration number (starting from 0) in the SART procedure. *λ* is the relaxation factor that plays a crucial role in both the reconstruction accuracy and the convergence speed. Apparently, *λ* represents the contribution of the methane absorption at the *j*-th grid to the integration of the *i*-th laser beam. Although the conventional SART algorithm can improve the reconstruction quality of gas distribution from incomplete beam projections, it is at the expense of sacrificing convergence speed [42]. Therefore, the fixed common constant *λ* of conventional SART is replaced by a dynamic relaxation function to change the iterative scale and acceleration convergence during the reconstruction process, named the dynamic relaxation and simultaneous algebraic reconstruction technique (DR-SART).

(11) $$\lambda^{\left(k\right)}=\omega \times \frac{l_{i,j}x_{j}^{\left(k\right)}}{\sum_{j=1}^{N}l_{i,j}x_{j}^{\left(k\right)}}$$

where *ω* is a constant in the iterative process.

In fact, the concentration of gas diffusion is always non-negative. Therefore, a non-negative constraint is set for the value of *x_j_*. Any negative concentration values that appear in the reconstruction result are set to zero and used as the initial value for the next iteration until the condition is satisfied. Convergence criterion is expressed by Equation (12), and reconstruction terminates when the methane concentration for each grid between two consecutive iterations becomes less than a preselected minimum *τ*.

(12) $$x_{j}^{\left(k+1\right)}-x_{j}^{\left(k\right)}\leq \tau \;\left(\tau =1\times 10^{-6}\right)$$

#### 2.2.2. Evaluation Indexes of Reconstruction Results

In order to evaluate the reconstruction quality of methane-diffusion distribution, the following six evaluation indexes are used to quantify the agreement between the original concentration field and the reconstructed result in this work. The normalized root mean square distance (*NRMSD*) sensitively reflects the situation where a few points in the reconstructed gas distribution map have larger errors. When there is a severe error between the reconstructed map and the original map at a certain point, the *NRMSD* will rapidly increase. Normalized average absolute distance (*NAAD*) will have increased emphasis on the importance of numerous small error points of the reconstructed gas distribution map than in the *NRMSD*. *NRMSD* and *NAAD* are always non-negative, and a value of zero indicates a perfect fit. The gas-distribution map similarity coefficient (*ε*) judges the distortion level of the reconstructed gas-distribution map, and its closeness to 1 reflects a consistency of contour structure information between the reconstructed image and the original image. *γ_a_* represents the average relative error of the reconstructed result. *γ_m_* represents the reconstruction error of maximum concentration between original gas distribution and the reconstructed result. *δ* represents the positional error of maximum concentration between original gas distribution and the reconstructed result. Their derivation expressed as follows:

(13) $$\begin{array}{l} NRMSD=\sqrt{\frac{\sum_{j=1}^{N}{\left(x_{orig,j}-x_{rec,j}\right)}^{2}}{\sum_{j=1}^{N}{\left(x_{orig,j}-{\bar{x}}_{orig}\right)}^{2}}},\\ NAAD=\frac{\sum_{j=1}^{N}\left|x_{orig,j}-x_{rec,j}\right|}{\sum_{j=1}^{N}x_{orig,j}},\\ \varepsilon =\frac{\sum_{j=1}^{N}\left|x_{orig,j}-{\bar{x}}_{orig}\right|\left|x_{rec,j}-{\bar{x}}_{rec}\right|}{\sqrt{\sum_{j=1}^{N}{\left(x_{orig,j}-{\bar{x}}_{orig}\right)}^{2}\sum_{j=1}^{N}{\left(x_{rec,j}-{\bar{x}}_{rec}\right)}^{2}}},\\ \gamma_{a}=\frac{1}{N}\sum_{j=1}^{N}\frac{\left|x_{orig,j}-x_{rec,j}\right|}{x_{orig,\max}-x_{orig,\min}},\\ \gamma_{m}=\frac{\left|x_{orig,\max}-x_{rec,\max}\right|}{x_{orig,\max}},\\ \delta =\frac{\sqrt{{\left(X_{orig,\max}-X_{rec,\max}\right)}^{2}+{\left(Y_{orig,\max}-Y_{rec,\max}\right)}^{2}}}{L_{\mathrm{diag}}} \end{array}$$

where *x_orig_*_,*j*_ and *x_rec_*_,*j*_ are the original and reconstructed concentrations in cell *j* of the gas-distribution map, *j* = 1, 2, …, *N*. *x_orig_* is average concentration of all cells in the original gas-distribution map. *x_rec_* is average concentration of all cells in reconstructed gas-distribution map. (*X_orig_*_/*rec*,max_, *Y_orig/rec_*_,max_) represent the position coordinates of the maximum concentration point in the reconstructed or original gas-distribution map. *L*_diag_ is the diagonal length of the measurement region.

## 3. Results and Discussions

### 3.1. Precision Estimation of TDLAS Telemetry Sensor

**Figure 4:**
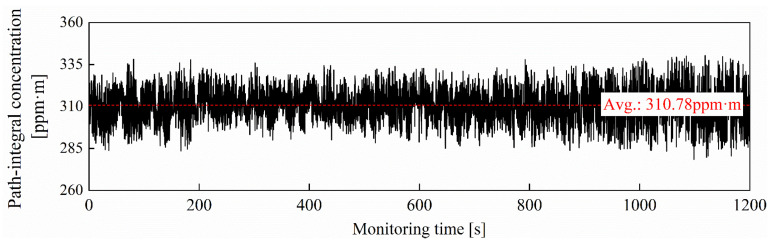
Methane path-integral concentrations measured by self-made TDLAS telemetry sensor during 1200 s monitoring period.

The detection precision of the TDLAS telemetry directly determines the sensing capability for trace-level concentrations of methane fugitive emissions. In order to investigate the limit-of-detection (LOD) performance of the methane laser telemetry system, time series measurements for the methane gasbag are performed in an empty corridor. A transparent balloon, filled with methane at a path-integral concentration of 300 ppm·m, is monitored at a detection distance of 10 m for 1200 s. The sampling interval for the TDLAS telemetry sensor is 0.2 s. The 6000 concentration data are successively recorded as shown in Figure 4.

**Figure 5:**
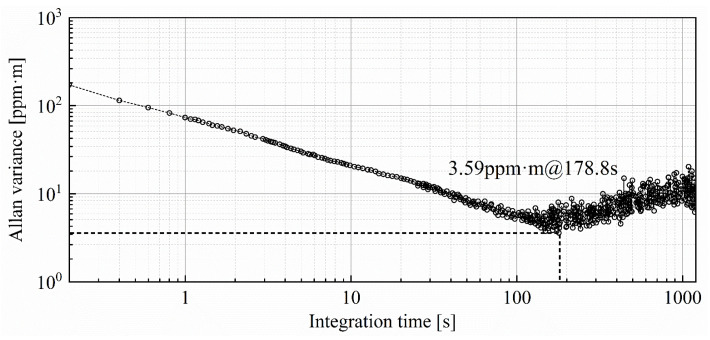
Methane path-integral concentrations measured by self-made TDLAS telemetry sensor during 1200 s monitoring period.

Allan variance analysis is the most common and widely used tool for estimating the minimum detection limit of TDLAS sensor systems [43]. The variance supplies detailed information, including the signal stability and noise characteristics of monitoring over different time scales. The Allan variance, depicted in Figure 5, is plotted as a function of integration time using the long-term monitoring data of methane path-integral concentrations. The minimum Allan variance value signifies the lowest level of system noise, indicating the state of maximum signal-to-noise ratio (SNR) for the sensor system. It indicates that the optimum integration time is 178.8 s, and the corresponding detection LOD is 3.59 ppm·m. When the integration time is within 178.8 s, the white noise dominates the noise source at this period. After exceeding the optimal integration time, further prolonging the integration time does not lead to additional enhancements in LOD. It should be noted that the minimum value of Allan variance is generally considered an estimated value of LOD; it does not directly measure the LOD itself. Allan variance analysis only provides a convenient means for estimating the LOD. In fact, the direct measurement of LOD requires other methods, such as the concentration step-down method, to determine the level at which the analyte concentration becomes undetectable.

### 3.2. Reconstruction Results Evaluation under Different Optical Path Modes

**Figure 6:**
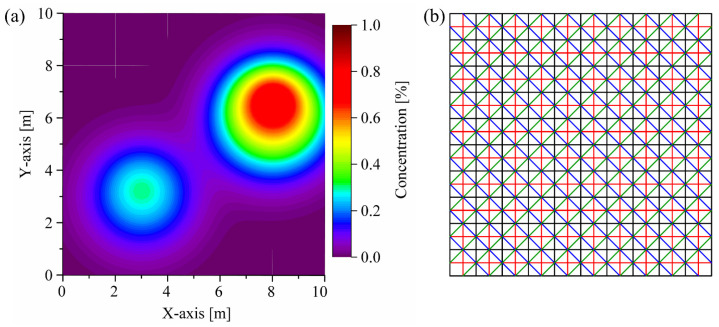
(**a**) Concentration map sample utilized for the optimization of parameter *ω*; (**b**) the four-angle parallel optical path layout is solely employed for parameter *ω* optimization study. The red lines represent the optical paths in the 0° and 90° directions. The blue lines represent the optical paths in the 45° direction. The green lines represent the optical paths in the −45° direction.

Optimizing the constant *ω* in the dynamic relaxation function is a crucial task for balancing convergence speed and reconstruction quality. As shown in Figure 6, a simplified concentration map on the horizontal plane is generated to explore the optimal selection of parameter *ω*, employing the Gaussian bimodal model for this investigation. Given no prior information of the target field, the reconstruction performance of the four-angle parallel optical path layout is demonstrated to be satisfactory [44,45].

**Figure 7:**
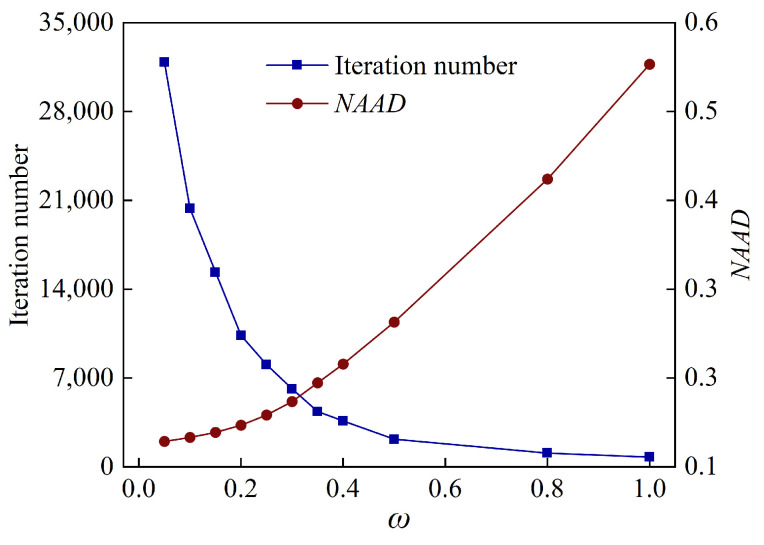
Effect of *ω* in dynamic relaxation function on reconstruction errors and convergence speed by DR-SART.

The iteration number is employed to characterize the convergence speed, while the *NAAD* is used to evaluate the reconstruction quality [46]. As shown in Figure 7, for a *ω* parameter of 0.05, the computation reaches 31,865 iterations. As *ω* increases, the computational load decreases rapidly. For a *ω* parameter of 0.3, the iteration count is reduced to 6149. Further increments in *ω* do not significantly reduce the computational load, and simultaneously, they lead to a degradation in the reconstruction quality due to over-adjustment during the iterative process. In this context, it is recommended to choose *ω* values within the range of 0.2 to 0.4, with *NAAD* corresponding to 0.029–0.031. In this work, a *ω* value of 0.3 is selected. The computational workload remains moderate under this choice, while ensuring a commendable reconstruction quality.

**Figure 8:**
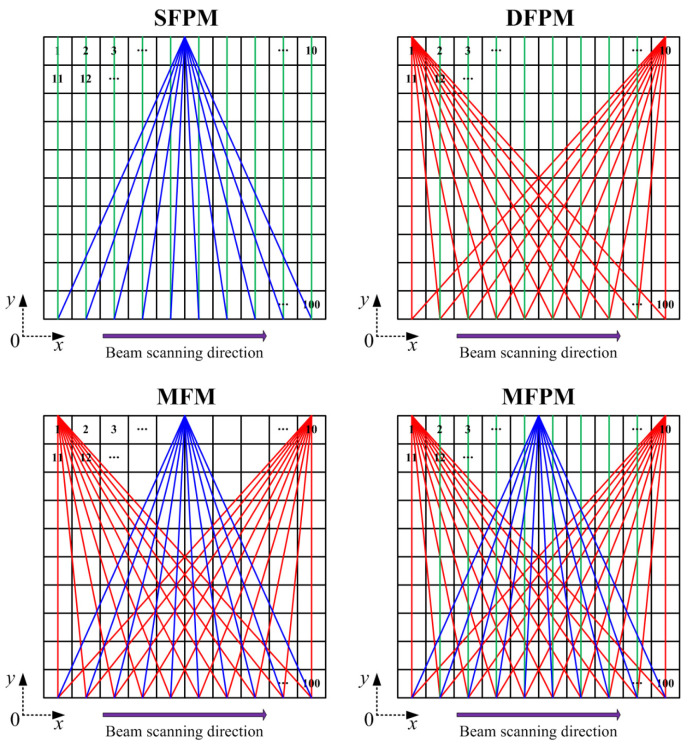
Laser-scanning coverage for the methane fugitive emissions scenario based on a 10 × 10 grid discretized area. Potential configurations of optical path projection: Single fan-beam combined with parallel-beam mode (SFPM), double fan-beam combined with parallel-beam mode (DFPM), multiple fan-beam mode (MFM), and multiple fan-beam combined with parallel-beam mode (MFPM). The red lines represent the fan-beam optical paths in the left and right sides. The blue lines represent the fan-beam optical paths in the middle. The green lines represent the parallel-beam optical paths in the vertical direction.

The optical path configuration affects the reconstruction accuracy. Generally, the reconstruction performance improves with an increasing number of laser beams overlapping in each grid. However, the increase in the projection numbers of the scanning laser beam entails a concomitant escalation in computational algorithm complexity and sensor quantity. To achieve a real-time monitoring of methane fugitive emissions distribution in the oil/gas facilities, a reduction in scanning time is imperative. Consequently, during the reconstruction process, a comprehensive consideration of factors such as sensor quantity, scanning-angle intervals, scanning-angle range, and scanning time, among others, must be undertaken to ascertain the optimal optical path configuration. In this work, vertical distribution reconstruction is investigated and only one laser telemetry sensor is employed for both mobile and rotational scanning. Mobile scanning generates a parallel-beam mode, while rotational scanning produces a fan-beam mode. The distinct coupling of these two beam modes enables the cross-coverage of discrete measurement-area grids, enhancing optical path density and spatial resolution. Optimization and selection analysis are applied to the four optical path-projection configurations in Figure 8.

**Figure 9:**
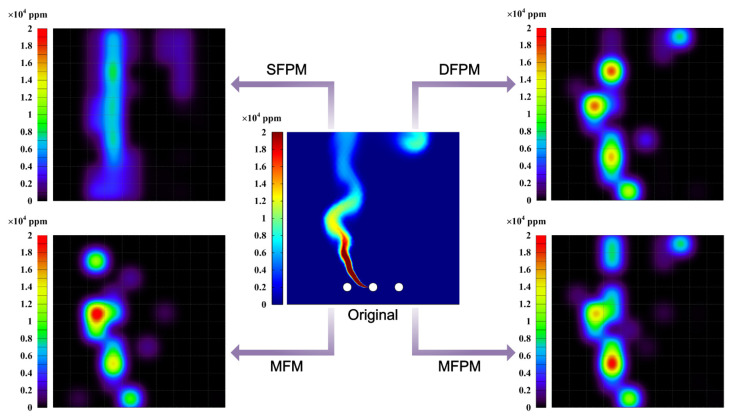
Reconstructed concentration maps of methane fugitive emissions’ diffusion distribution obtained through four optical path-projection configurations: SFPM, DFPM, MFM, and MFPM.

**Figure 10:**
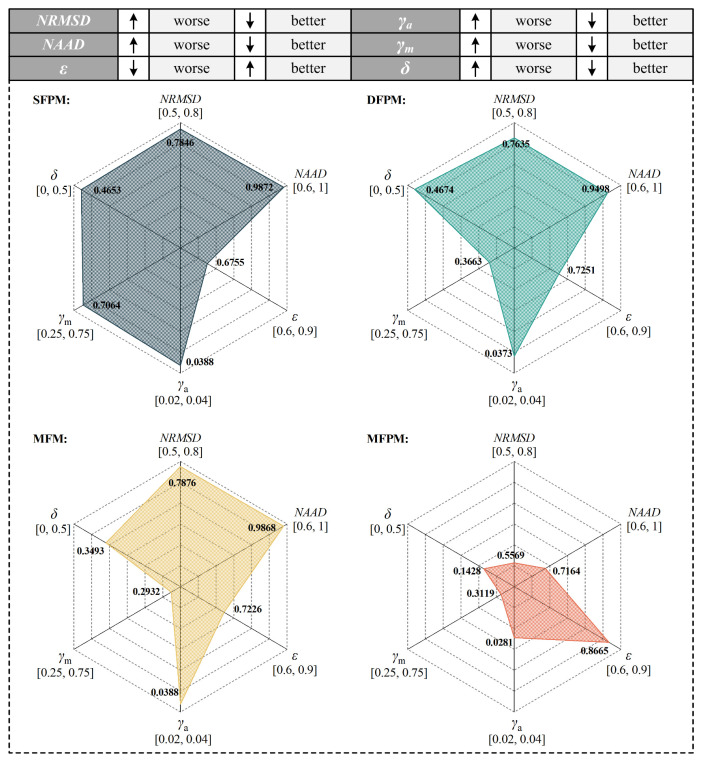
The evaluation indexes for reconstruction effects under different optical path configurations. The radial axis of each index is labeled with a mathematical closed interval [a, b] to represent different scales and the division of ticks on each axis is uniform. And, the scale of each index radial axis is consistent between the different optical path-projection configurations.

In order to evaluate the reconstruction performance of these optical path-projection configurations, the distribution map of methane fugitive emissions in the typical oil/gas-gathering and -boosting facilities are used as testing objects. As a simple and feasible scheme, the methane-diffusion distribution is obtained by using a multi-physics coupling finite element method [47,48,49]. Using the validated numerical codes, including Reynolds-averaged Navier-Stokes (RANS) equations, a standard two-equation *k*-*ε* model, and a mixture-averaged diffusion model, we conducted methane fugitive emissions simulations for one leakage source location in a natural-gas-gathering pipeline. The projection of the scanning laser beam used for methane-distribution reconstruction lies on the simulated concentration field section. The one-dimensional line-integral method is used to calculate the path-integral concentration *Y* of each laser beam. Based on the path-integral concentrations, the reconstructed results of these four optical path-projection configurations are obtained and compared, as shown in Figure 9. And, the evaluation indexes for reconstruction effects under different optical path configurations are shown in Figure 10.

The original gas-distribution map in Figure 9 provides a typical sample of methane plume dispersion, encompassing the plume column on the left side of the nephogram and the diffusive cloud at the top right of the nephogram. The difference between the SFPM and DFPM optical path-projection configurations lie in the number of fan-beams superimposed on the parallel-beam and the projection angle. While both can reconstruct plume columns and diffusive clouds, a quantitative analysis indicates a slightly superior reconstruction quality for DFPM. However, the major drawback of DFPM is that the reconstructed concentration gradient shows a disordered mismatch compared to the original map. This also means that, for the reconstruction of the vertical distribution of gas-plume diffusion, simply increasing the number and angle of laser beam projections has limited contribution to improving the reconstruction quality. The MFM configuration replaces the parallel-beam with a fan-beam light path based on DFPM. And, the number of laser beam projections for both optical path configurations is equal, with each comprising 30 laser beams. Due to the lack of beam projection in some grids of the reconstruction region, there is a manifestation of missing information in the diffusive cloud at the top right of the MFM reconstruction map. However, its maximum concentration reconstruction error *γ*_m_ is 0.2932, which is significantly improved compared to SFPM and DFPM. The *γ*_m_ cannot be solely taken as an index to explain the reconstruction quality. It must be complemented by the maximum concentration positional error δ. As shown in the results, although the *γ*_m_ of MFM is the smallest among the four optical path-projection configurations, the position of the maximum concentration point in the reconstructed map does not match the original map. Conversely, the reconstructed map obtained through MFPM is closest to the original map in terms of maximum concentration position, i.e., *δ* = 1.01 m, and the *γ*_m_ is also within an acceptable range. Meanwhile, the MFPM configuration combines parallel-beams and three types of fan-beams for joint multiplexing, ensuring that majority grids can receive multiple and multi-angle laser beam projections. As shown in Figure 10, the reconstruction performance of the MFPM configuration is superior, and its reconstructed average relative error *γ*_a_ is 0.0281. In comparison to the other three optical path configurations, the *NRMSD* and *NAAD* of the MFPM configuration exhibited reductions of 27.7% and 25.8%, respectively, while *ε* increased by approximately 22.4%. In practical applications, TDLAS telemetry sensors will be employed to obtain path-integral concentrations, exhibiting inherent signal noise and errors. The presence of measurement noise and errors may result in an escalation of the reconstruction error. Additional experimental studies are necessary to evaluate the reconstruction performance of the MFPM configuration. Moreover, owing to the fixed response time of the TDLAS sensor, an increased number of optical paths necessitate additional time for scanning the measurement region.

### 3.3. Experimental Analysis of Methane Vertical Distribution Mapping

**Figure 11:**
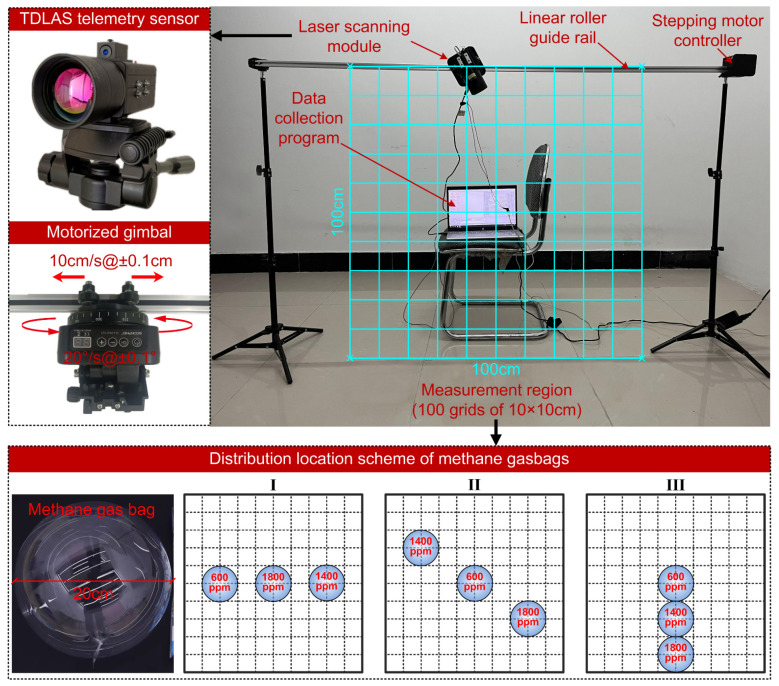
Experimental platform of methane-concentration-map reconstruction based on scanning laser telemetry path-integral sensing.

In order to demonstrate the reliability of MFPM optical path configuration, a scanning-laser-reconstruction experiment for different methane concentration distributions is implemented. As shown in Figure 11, the entire measurement region is a vertical cross-section of 100 × 100 cm relative to the ground, which can be pre-divided into 100 grids of 10 × 10 cm each. It is enclosed by the horizontal linear roller guide rail and the tripods supporting the ends of the guide rail. The mobile slider suspended on the guide rail is controlled for its horizontal motion by a stepper motor, with a maximum translational speed of 20 cm/s and a positioning accuracy of ±0.1 cm. The laser scanning module is comprised of the self-made TDLAS telemetry sensor mounted on a motorized gimbal, and the entire module is securely fastened to the mobile slider using M4 screws. The motorized gimbal permits a 360° rotation around its axis, with a rotational speed ranging from 0.1°/s to 60°/s and a positioning accuracy of ±0.1°. The mobile slider and motorized gimbal, through the execution of pre-compiled PLC logic instructions, regulate the TDLAS telemetry sensor to accomplish the MPFM laser-scanning scheme. The scanning duration encompasses the time consumed by the rotational motion and translational motion of the TDLAS telemetry sensor, and the measurement time required for obtaining the path-integral concentration along each laser beam. For the TDLAS telemetry sensor we provide, the concentration measurement frequency is set to 50 Hz, while the measurement time of 10 s is allocated for each optical path. When the rotational speed of motorized gimbal and the translational speed of mobile slider are set at 10°/s and 10 cm/s, respectively, the total scanning duration of MFPM configuration amounts to 411.29 s. To construct methane distribution with different forms within the measurement region, the transparent gas bags with a diameter of 20 cm, filled with methane gas samples of varying concentrations (600 ppm, 1400 ppm, and 1800 ppm), are utilized. The methane gas bags are suspended in the measurement region using fishing lines and deployed according to the distribution-location scheme outlined in Figure 11. The influence of the dynamic evolution of gas diffusion on the reconstruction results is not considered in this experiment. In real outdoor environments, continuous leaks or varying environmental wind speeds can lead to spatiotemporal variability in the gas-concentration field. However, it should be noted that this experiment focuses more on preliminary work, including the feasibility verification of the overall collaboration of the optical path configuration, reconstruction algorithm, and telemetry sensing systems in gas-distribution mapping.

**Figure 12:**
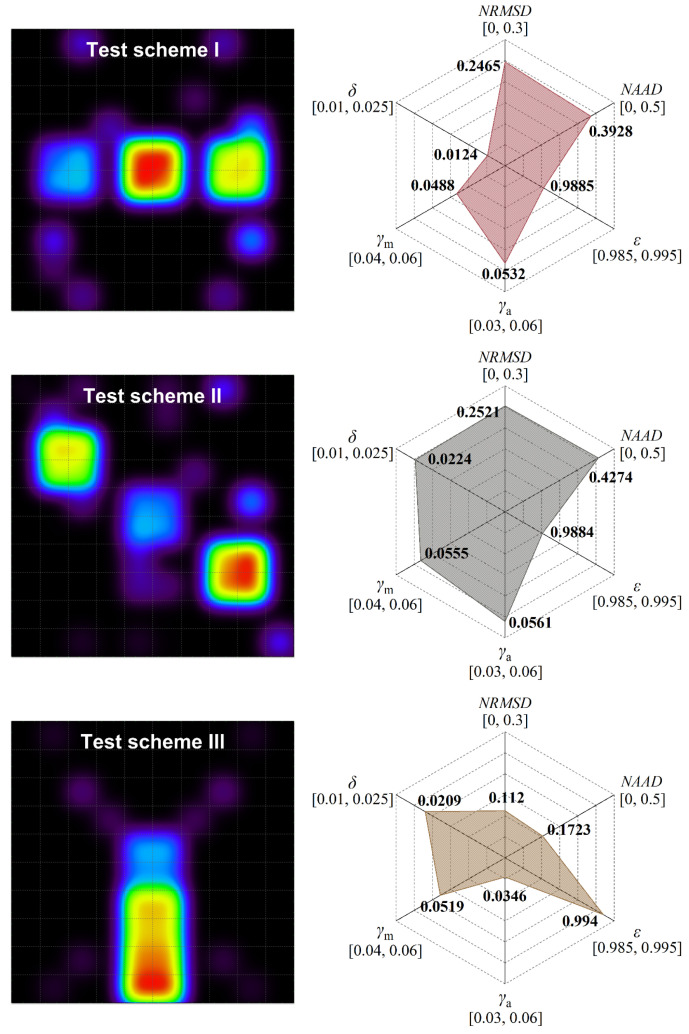
Gas-distribution-map reconstruction results and the corresponding quantitative evaluations for different methane gas bag-layout schemes.

As shown in Figure 12, the concentration distributions in the experimental reconstruction maps are consistent with the three arrangements of methane gas bags, which confirms that the MFPM optical path configuration has considerable tomographic reconstruction performance. It is noteworthy that the methane gas bags employed in the experiment are spherical; however, the methane distribution in the reconstruction maps appears to be square. The primary reason for this discrepancy lies in the insufficient grid division of the measurement region, resulting in a decline in spatial resolution. While ensuring the reconstruction accuracy, enhancing the grid resolution must be accompanied by an increase in optical path projection data. Failure to do so may lead the algorithm into a state of local minima or under-learning. In practical applications, this may not be a cost-effective choice. In addition, unexpected concentration artifacts appeared at some positions in the reconstructed maps. The occurrence of this phenomenon is attributed to the sparsity of projection data in some grids, thereby inducing an augmentation of ill-posed-ness in the algorithm-inversion process. Furthermore, in the measurement mode of non-cooperative open paths, the laser beam may undergo multiple absorptions or scatterings via methane gas bags during its propagation. This gives rise to a discrepancy between the measured light intensity at specific positions and the actual conditions, resulting in the generation of spurious artifacts. Based on the six-index analysis, a quantitative evaluation is conducted on the reconstruction qualities of different methane gas bag-layout schemes. The similarity coefficient ε between the reconstruction maps of these three testing schemes and the true concentration distribution exceeds 0.98. Compared to the numerical simulation map, the ε index experiences a significant improvement. This is primarily attributed to the lower complexity of the methane gas bag distribution in the experimental setup compared to the numerical simulation results. The ordering of concentration-distribution results is a diminished influence of non-linear effects on the reconstruction algorithm operation. In measurement scenarios involving the stable settling of leaked pollutants, such as underground gas valve wells or natural gas galleries, the laser scanning reconstruction of gas distribution maps may yield results closer to actual conditions. Compared to the other two test schemes, the reconstruction map of scheme III exhibits smaller values for the index *NRMD*, *NAAD* and *γ*_a_, indicating its superior overall reconstruction accuracy. Additionally, the *γ*_m_ values for all three reconstruction outcomes are consistently around 0.05, with the index *δ* falling within the range of 0.01 to 0.025. This suggests that the proposed approach demonstrates favorable performance in predicting and localizing concentration peaks. However, it must be noted that there are significant differences between indoor experimental details and field scenarios. Our future work will focus on the measurement of dynamic methane-release processes.

In order to investigate the influence of scanning duration on reconstruction accuracy, the distribution location scheme II of methane gas bags is selected as the test subject. By altering the measurement time along each optical path, different scanning durations are obtained, while keeping the rotational speed of the motorized gimbal and the translational speed of the mobile slider. The specific configuration parameters for scanning duration are presented in Table 1.

**Figure 13:**
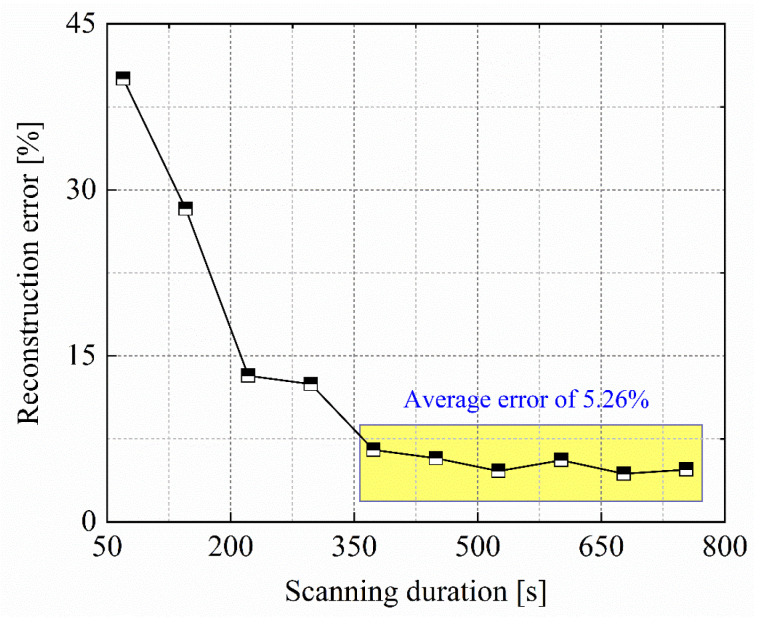
Effect of scanning duration on reconstruction error of methane vertical distribution.

As shown in Figure 13, error analysis is conducted based on the reconstructed concentration at the center point of the 1400 ppm methane gas bag. With the extension of scanning duration, the reconstruction error gradually diminishes, demonstrating a convergent trend and achieving regional stability. Upon each optical path switch achieved through the motorized gimbal or mobile slider, persistent minor mechanical vibrations are inevitably generated. When the measurement time of the TDLAS telemetry sensor on each optical path is relatively short, the measurement errors introduced by mechanical vibrations become significant. A viable method to mitigate such errors involves extending the measurement time for individual optical paths to acquire more stable path-integral concentration values. In this experiment, the indication sampling time of the TDLAS telemetry sensor is set at 0.2 s. When the measurement time at each scanning position exceeds 9 s, corresponding to a scanning duration exceeding 300 s, the reconstruction error can generally be maintained within 5.26%. Further extension of the scanning duration does not yield higher reconstruction accuracy, and is primarily constrained by the inherent detection performance of the TDLAS telemetry sensor.

## 4. Conclusions

In this work, we developed a TDLAS telemetry sensor with an excellent sensitivity and an improved tomographic algorithm for the vertical distribution mapping of methane fugitive emissions from oil/gas facilities. A six-indexes system is employed for the reconstruction performance analysis for four potential optical path-projection configurations, and the corresponding validation experiment is conducted. Relative to the other three optical path configurations, the MFPM configuration demonstrated decreases of 27.7% and 25.8% in *NRMSD* and *NAAD*, respectively, with an approximately 22.4% increase in ε. For the different methane gas bag layout schemes, the reconstruction errors of maximum concentrations are consistently around 0.05, with the positional errors of maximum concentration falling within the range of 0.01 to 0.025. Furthermore, considering the trade-off between scanning duration and reconstruction accuracy, it is advisable to extend the sensor measurement time on a single optical path appropriately to alleviate the influence of mechanical vibrations caused by scanning motion.

The proposed methodology underwent testing on a controlled indoor experimental platform. In practical applications, on-site environments may present considerable complexity, particularly regarding instantaneous variations in concentration distribution caused by environmental wind speeds on the reconstruction outcomes. This aspect constitutes a focal point for our future research endeavors. Additionally, more advanced technologies such as laser scanning radars and augmented reality have the potential to facilitate the wearable monitoring of methane-emission-distribution mapping.

## Figures and Tables

**Table 1:** The specific configuration parameters for scanning duration.

Measurement Time [s]	Scanning Duration [s] *
1	69.3
3	145.3
5	221.3
7	297.3
9	373.3
11	449.3
13	525.3
15	601.3
17	677.3
19	753.3

* The scanning duration is the total of measurement time, rotation time, and translation time. Rotational speed of motorized gimbal is set 10°/s, corresponding to a total rotation time of 22.29 s. Translational speed of mobile slider is set 10 cm/s, corresponding to a total translation time of 9 s.

## Data Availability

The data that support the findings of this study are available from the corresponding author upon reasonable request.
